# Cytotoxic T-lymphocyte antigen 4 and programmed cell death ligand 1 blockade increases the effectiveness of interleukin-15 immunotherapy in a bovine leukemia model

**DOI:** 10.14202/vetworld.2024.2096-2103

**Published:** 2024-09-15

**Authors:** Kanatbek Mukantayev, Kanat Tursunov, Zhansaya Adish, Darkhan Kanayev, Laura Tokhtarova, Malika Nurtleu, Bisultan Abirbekov

**Affiliations:** 1 Laboratory of Immunochemistry and Immunobiotechnology, National Center for Biotechnology, 010000, Astana, Kazakhstan; 2 Department of Natural Sciences, L.N. Gumilyov Eurasian National University, 010008, Astana, Kazakhstan

**Keywords:** bovine interleukin-15, bovine leukemia virus, cytotoxic T-lymphocyte antigen 4, monoclonal antibody, programmed cell death ligand 1

## Abstract

**Background and Aim:**

Bovine interleukin 15 (bIL15) is a potential immunotherapy that can block the spread of bovine leukemia virus (BLV). However, immune checkpoints that maintain body homeostasis may reduce their effectiveness. Thus, an analysis of the effectiveness of bIL15 while blocking negative immune regulators is necessary. We aimed to obtain recombinant bIL15 (rbIL15) and determine its percentage using monoclonal antibodies against bovine cytotoxic T-lymphocyte antigen 4 (CTLA-4) and programmed cell death ligand 1 (PD-L1). To achieve this goal, peripheral blood mononuclear cells (PBMCs) from healthy and BLV+ cattle were treated with bIL15 using a CTLA-4– and PD-L1–blocking algorithm.

**Materials and Methods:**

The codon-optimized *bIL15* gene was synthesized under *de novo* conditions using polymerase chain reaction (PCR). The synthesized gene was cloned into pET28 and transformed into electrocompetent *Escherichia* *coli* BL21 cells; rbIL15 was purified using metal affinity chromatography and analyzed using sodium dodecyl-sulfate–polyacrylamide gel electrophoresis (SDS-PAGE) and western blotting. The expression of the *Bcl2*, *STAT3*, and *STAT5* genes was studied using qualitative PCR. An enzyme-linked immunosorbent assay (ELISA) was used to analyze interferon (IFN)-γ production by rbIL15-treated mononuclear cells.

**Results:**

Analysis of rbIL15 using SDS-PAGE and western blotting revealed a specific product weighing 24 kDa. The optimal conditions for rbIL15 induction were 0.2 mM isopropyl-β-D-1-galactopyranoside and 37°C. When rbIL15 was added to PBMCs from healthy cattle, the *Bcl2*, *STAT3*, and *STAT5* genes were expressed. ELISA of the culture medium of rbIL15-treated PBMCs revealed IFN-γ production. When PBMCs from healthy cows were treated with rbIL15, CTLA-4, and PD-L1 blockade together, they did not produce more IFN-γ than the rbIL15 group. Using PBMCs from BLV+ cattle, combination treatment increased IFN-γ production.

**Conclusion:**

The biological activity of rbIL15 is characterized by the induction of transcription factors and the production of IFN-γ. Using rbIL15 with CTLA-4 and PD-L1 blockade in PBMCs from healthy and BLV+ cows led to the production of a transcription factor and cytokine. The results demonstrate the possibility of using this method to improve immunity and immunological memory in patients with chronic viral infections.

## Introduction

The causative agent of bovine leukemia is a virus belonging to the family *Retroviridae*, which is similar to human and simian T cell leukemia viruses. Despite ongoing research efforts to improve methods for preventing bovine leukemia, there is a growing need to study the immune response mechanisms against bovine leukemia virus (BLV) [1]. In bovine leukemia, disruption in cytokine production, decrease in proliferation, and increase in effector T cell apoptosis are observed [2]. Cytokine production is an essential function of the immune system and influences the growth and activity of various immune cells, as well as the strength and duration of the immune response. A link was observed between higher levels of transforming growth factor (TGF)-β and interleukin (IL)-10 and lower levels of interferon (IFN)-γ, IL-12, IL-2, and IL-4 levels in BLV-infected cows. Increased IL-10 expression is associated with increased regulatory B and T cell counts, leading to immunosuppression [3–5].

The cytokines IL-12, IL-15, and IL-7 play essential roles in the proliferation of effector T cells and mediate long-term immunological response. The expression of these cytokines contributes to an increase in the concentration of cluster of differentiation 8 (CD8+) T cells in immunological memory and its maintenance. Several studies by Vijay *et al*. [6], Zhang *et al*. [7], and Ara *et al*. [8] demonstrated the active and selective effect of IL-15 on CD8+ T cells. *Ex vivo* and *in vivo* administration of IL-15 increased the proliferation of exhausted T cell precursors. In addition, IL-15 increased the functionality of natural killer (NK) and CD8+ memory T cells in lymphoma patients, thereby improving immunity [9, 10]. The immune system is activated by IL-15, which in turn activates the Jak, STAT3, STAT5, Bcl-2, MCL-1, and c-Myc signaling pathways, helping NK cells remain alive. STAT3 activates the transcription of IFN-γ genes and other molecules responsible for the effector functions of NK cells [11, 12]. In the case of solid tumors in mouse models, IL-15 was more effective than anti-programmed death-1 (PD-1) antibodies. *In vitro*, IL-15 induced the proliferation of CD28–PD-1 + CD8 + tumor-infiltrating lymphocytes (TIL) cells more effectively than anti-PD-1 antibodies. According to the author, anti-PD-1 antibodies reacted and activated only tumor antigen–specific cells, whereas IL-15 was activated regardless of antigen specificity. When IL-15 and anti-PD-1 antibodies are combined, they slow tumor growth because of CD8+ T cells [13]. Combining IL-15 therapy with anti-cancer monoclonal antibodies (mAbs) has been shown to improve their antibody-dependent cellular cytotoxicity [14].

Given its positive effects on NK and CD8+ memory T-cell proliferation, IL-15 has potential in treating bovine leukemia. One promising area of therapy for bovine leukemia is the combination of IL-15 with immune checkpoint blockade. When IL-15 anti–programmed cell death ligand 1 (PD-L1) and anti–cytotoxic T-lymphocyte antigen 4 (CTLA-4) antibodies were used together in mouse tumor models, the latter were able to survive for a longer time [15].

The purpose of this study was to determine the effect of combined blockade of PD-L1 and CTLA-4 receptors with recombinant bovine IL 15 (rbIL15) on the induction of immunity in cattle *in vitro*. This study investigated the biological activity of rbIL15 optimized for *Escherichia* *coli* expression. The results of activating peripheral blood mononuclear cells (PBMC) in healthy and asymptomatic leukemic cows using rbIL15 along with anti-PD-L1 and anti-CTLA-4 antibodies are also presented.

## Materials and Methods

### Ethical approval

The LLP “National Center for Biotechnology” Ethics Committee approved the study (00013497). Blood samples were collected by a trained person without harming or causing unnecessary stress to the animals.

### Study period and location

The study was conducted from July 2023 to April 2024 at the National Center for Biotechnology (Astana, Kazakhstan). Whole blood samples were collected from animals on farms and farmsteads in Akmola during routine veterinary examinations.

### Bacterial and plasmid strains

Laboratory strains of *E. coli* DH5α and BL21 (DE3) (Novagen, Cambridge, UK) were used to obtain rbIL15. For cloning and production of a construct based on an expression vector, plasmids pGEM-T (Promega, Madison, WI, USA) and pET28 (Novagen) were used for cloning and production of a construct based on an expression vector, respectively.

### Gene synthesis, cloning, and rbIL15 expression

The amino acid sequence of bIL-15 was obtained from the PubMed National Center for Biotechnology Information reference sequence: AAA85130.1. A Verso complementary DNA (cDNA) synthesis kit (ThermoFisher Scientific, Vilnius, Lithuania) was used for the synthesis of сDNA. Vector NTI Advance 11.5.0 (Invitrogen, Madison, WI, USA) was used for reverse translation and codon optimization of the amino acid sequence for expression in *E. coli*. The corresponding oligonucleotides were designed using DNAWorks (http://helixweb.nih.gov/dnaworks/). Sequence synthesis was conducted *de novo* using a two-round polymerase chain reaction (PCR) test. The PCR products were purified, cloned into pGEM-T, and sequenced. The synthesized gene was recloned into pET28 using EcoRI/XhoI (Thermo Fisher Scientific, Waltham, MA, USA) restriction sites and transformed into competent *E. coli* BL21(D3) cells.

The selected transformed *E. coli* BL21(D3) cells were cultured in 250 mL of lysogeny broth containing ampicillin and incubated in a shaker at 160 rpm and 37°C. During the logarithmic phase, 0.2 mM isopropyl-β-D-1-galactopyranoside (IPTG) was added, and incubation was continued for 16 h at 37°C and 160 rpm. Cells were harvested through centrifugation at 6000× *g* for 7 min at 4°C. The cell pellets were resuspended in lysis buffer and lysed using an ultrasonic disintegrator. After centrifugation of disintegrated cells, the pellet was resuspended in a buffer containing 1-M urea, incubated for 30 min at 23°C, and centrifuged at 20,000× *g* for 30 min. The pellet was dissolved in a buffer containing 8 M urea and sonicated. The protein was further purified using Histrap columns (GE Healthcare, Uppsala, Sweden). The columns were rinsed and equilibrated with a buffer containing 8-M urea. For the refolding of bound proteins, a linear gradient of 8–0 mol/L urea was formed in the column at a total volume of 30 mL and a flow rate of 0.5 mL/min. The refolded protein was eluted using a linear imidazole gradient.

### Western blot

Recombinant proteins were separated using 12% sodium dodecyl-sulfate–polyacrylamide gel electrophoresis (SDS-PAGE) and transferred onto nitrocellulose membranes. The membrane was first blocked by incubation in EveryBlot Blocking Buffer (Bio-Rad Laboratories, Tokyo, Japan) at 23°C for 5 min and then rinsed 3 times in phosphate-buffered saline (PBS) with 0.1% Tween 20 (PBS-Tw) buffer at pH 7.4. After rinsing, the membrane was incubated with anti-6His-tag mAbs and horseradish peroxidase (Thermo Fisher Scientific) diluted at a ratio of 1:5000 for 1.5 h at 23°C. The membrane was rinsed 3 times with PBS-Tw and 3 times with PBS without Tween 20 and visualized using 4-chloro-1-naphthol (Sigma-Aldrich, St. Louis, MO, USA).

### Nanoscale liquid chromatography with tandem mass spectrometry (LC-MS/MS)

Recombinant proteins were separated using 12% SDS-PAGE and stained with Coomassie blue. Further sample preparation was conducted as described by Adish *et al*. [16]. The peptides were separated using high-performance liquid chromatography and analyzed using LC-MS/MS. Mascot software (https://www.matrixscience.com/) was used to search for spectra in the SwissProt database (https://www.uniprot.org/).

### rbIL15 activity

PBMCs were isolated from the blood of healthy cattle using LymphoPrep™ Tube (Progen Biotechnik GmbH, Heidelberg, Germany) and resuspended in Roswell Park Memorial Institute (RPMI-1640) medium supplemented with 10% fetal bovine serum (10^6^ cells/mL) were transferred into a 6-well plate and incubated with 1 μg/mL rbIL15. After 12 h of incubation, cells were harvested, and messenger RNA (mRNA) was isolated using Donald C. Rio’s protocol [17]. The obtained cDNA was analyzed through PCR using primers specific for the *Bcl2*, *STAT3*, and *STAT5* genes. PCR with bovine-specific primers (Table-1), designed according to Vijay *et al*. [6], was used to determine the expression of *Bcl2*, *STAT3*, and *STAT5*.

**Table-1:** List of primers specific to Bcl2, STAT3, and STAT5.

No.	Name	Primer sequence 5′-3′	Length
1	Bcl2 Forward	AGCGTCAACCGGGAGATGT	19
2	Bcl2 Reverse	TAGGGCCATACAGCTCCAC	19
3	STAT5A Forward	GTACCCACAGAACCCTGAC	19
4	STAT5A Reverse	AGAGAGGGCTCCAGACTGT	19
5	STAT3 Forward	CAATACCATTGACCAGCCGAT	21
6	STAT3 Reverse	GAGCGACTCAAACTGCCCT	19

### Activation of bovine PBMCs by rbIL15 and CTLA-4 and PD-L1 blockade

To study the synergistic blockade of CTLA-4 and PD-L1, PBMCs were isolated from healthy and BLV-infected cattle with LymphoPrep™ (PROGEN Biotechnik GmbH). We cultured 10^6^ cells/mL in RPMI-1640 medium (Sartorius, Israel) supplemented with 10% fetal bovine serum (Biological Industries, Israel) in 6-well plates (Corning Inc., Costar) at 37°C in an atmosphere containing 5% CO_2_. To activate PBMCs, cells were incubated with 1 μg/mL rbIL15. To block CTLA-4 and PD-L1 receptors, cells were cultured with anti-PD-L1 (mouse immunoglobulin [Ig]G1) and anti-CTLA-4 (mouse IgG1) mAbs in the supernatant of fetal lamb kidney (FLK) cells. As a negative control, mouse IgG was used in the presence of heat-inactivated supernatants from FLK cell cultures. The culture medium was stored at −20°C for IFN-γ determination through enzyme-linked immunosorbent assay (ELISA). The bovine IFN-γ ELISA kit (Abcam, Boston, MA, USA) was used to determine IFN-γ concentration according to the manufacturer’s protocol. The PBMC blockade assay and IFN-γ determination were conducted as described by Okagawa *et al*. [18].

### Statistical analysis

GraphPad Prism 9.3.1 (GraphPad Software, San Diego, CA, USA) was used to construct the plots and perform statistical analysis. The differences between the two groups were examined using Student’s t-test. The statistical tests performed and their corresponding p-values are listed in the figure legends. p < 0.05 was considered statistically significant.

## Results

### Gene synthesis, cloning, and rbIL15 expression

**Figure-1:**
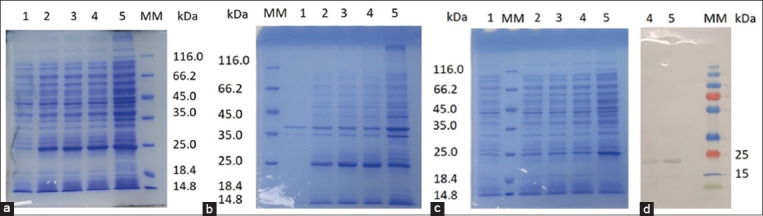
SDS-PAGE and western blot analyses of *rbIL15* expression. (a) Total, (b) pellet, (c) super, and (d) western blots. Lane 1: *Escherichia coli* culture without expression; Lane 2: Protein expression in 2 h; Lane 3: Protein expression in 4 h; Lane 4: Protein expression in 6 h; Lane 5: Protein expression in 12 h; Lane MM: Molecular markers. SDS-PAGE=Sodium dodecyl-sulfate–polyacrylamide gel electrophoresis, *rbIL15*=Recombinant bovine interleukin 15.

After cloning the synthesized gene into an expression vector, the resulting pET28/rbIL15 construct was transformed into BL21 bacterial cells. Cells were then cultured in a medium supplemented with 0.2 mM IPTG to determine gene expression, and samples were collected at various time points. SDS-PAGE analysis showed that recombinant protein expression occurred after 2 h and increased slightly with further incubation. The molecular weight of the rbIL15 protein was 24 kDa, which corresponded to the theoretical value (Figure-1). The bulk of the rbIL15 protein was localized in an insoluble form in inclusion bodies (Figure-1b). Western blot analysis using His-tagged mAb revealed the presence of a protein with a molecular mass of 24 kDa, which corresponded to the molecular mass of rbIL15 (Figure-1d).

### Optimization of the protein isolation, purification, and characterization of rbIL15

**Figure-2:**
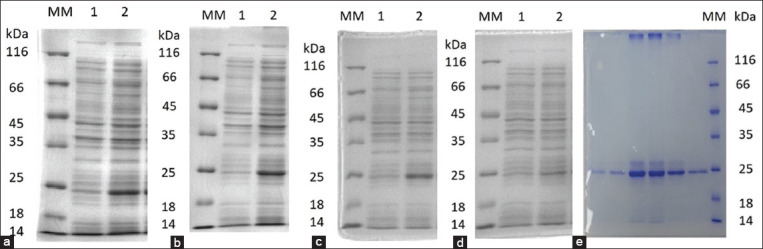
SDS-PAGE optimization and *rbIL15* purification. (a) 0.2 mM IPTG, (b) 1 mM IPTG, (c) 37°C, (d) 25°C, and (e) purified rbIL15. Lane 1: Without expression; Lane 2: With expression; Lane MM: Molecular markers. IPTG=Isopropyl-β-D-1-galactopyranoside, SDS-PAGE=Sodium dodecyl-sulfate–polyacrylamide gel electrophoresis, *rbIL15*=Recombinant bovine interleukin 15.

Because of this study, the isolation and purification of rbIL15 from inclusion bodies were optimized. Bacterial cells were cultured at IPTG concentrations of 0.1, 0.25, 0.5, and 1 mM at 25°C and 37°C. Increasing the IPTG concentration from 0.2 mM (Figure-2) to 1 mM (Figure-2) did not significantly affect rbIL15 production. The optimal cell culture temperature for rbIL15 production was 37°C (Figure-2b). Lowering the cultivation temperature to 25°C sharply reduced the productive properties of the producer strains (Figure-2d). To solubilize and isolate rbIL15 from inclusion bodies, the cell pellet was treated with 8 M urea before sonication. Because of purification and refolding using nickel-nitriloacetic acid (Ni–NTA) chromatography, purified rbIL15 was obtained (Figure-2e).

**Figure-3:**
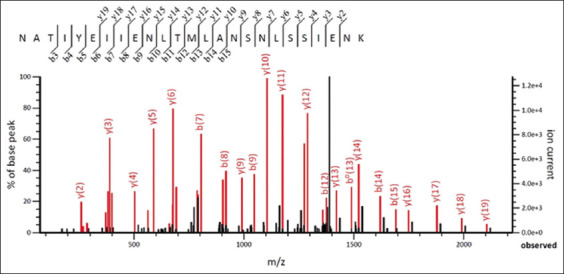
Tandem mass spectrometry of fragmented rbIL15. rbIL15=Recombinant bovine interleukin 15.

The spectrum of rbIL15 ions from LC-MS/MS was compared with that of the Mascot database, and the spectrum matched that of bIL-15 ions (Figure-3). In the Mascot database, one of the ion spectras matched the amino acid sequence NATIYEIIENLTMLANSNLSSIENK of bIL-15 (Score 20685).

### Biological properties of rbIL15

**Figure-4:**
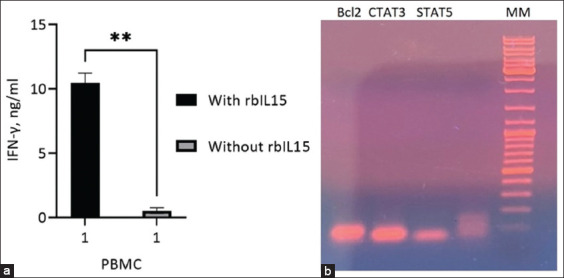
Biological activity of rbIL15. Concentration histograms of (a) IFN-γ and (b) PCR transcription factors of bovine PBMCs cultivated with and without *rbIL15*. rbIL15=Recombinant bovine interleukin 15, IFN=Interferon, PCR=Polymerase chain reaction, PBMCs=Peripheral blood mononuclear cells.

The biological activity of rbIL15 was determined by stimulating bovine PBMCs and detecting the mRNA of factors such as Bcl2, STAT3, and STAT5 [6, 19]. cDNA synthesized from mRNA was analyzed using PCR. The culture medium of bovine PBMCs stimulated with rbIL15 protein was examined for IFN-γ production using ELISA. The addition of rbIL15 increased both IFN-γ production (up to 12 ng/mL) and the expression of bcl2, STAT3, and STAT5 in bovine PBMCs (Figure-4). Electrophoresis of the PCR products showed that rbIL15 most actively induced the expression of *bcl2* and *STAT3*. The PCR product of STAT5 was two times smaller than those of bcl2 and STAT3.

### Efficacy of rbIL15 against CTLA-4 and PD-L1 mAbs *in vitro*

**Figure-5:**
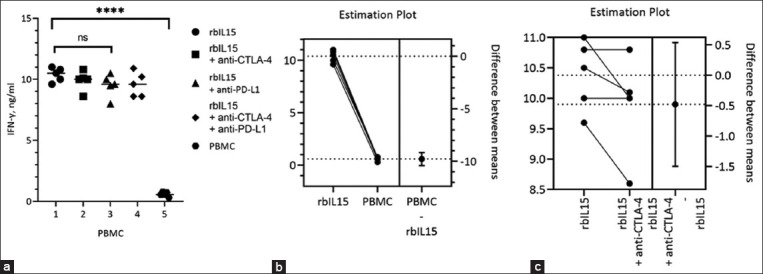
Effect of combining rbIL15 with CTLA-4 and PD-L1 blockade on healthy cattle PBMCs. (a) Enzyme-linked immunosorbent assay of culture media supernatant to determine IFN-γ concentration. (b and c) Estimated plot of differences between groups. Student’s t-test was used for comparisons. At p < 0.0001, differences were considered significant. rbIL15=Recombinant bovine interleukin 15, IFN=Interferon, PBMCs=Peripheral blood mononuclear cells, CTLA-4=Cytotoxic T-lymphocyte antigen 4, PD-L1=Programed cell death ligand 1.

To study the effects of the combined application of rbIL15 and blocking of CTLA-4 and PD-L1 receptors with mAbs, PBMCs from five healthy cows were used. Cells were cultured in a medium supplemented with rbIL15 and mAbs against bovine CTLA-4 and PD-L1, both individually and in combination. IFN-γ cytokine production in cultured PBMCs was analyzed using ELISA (Figure-5).

IFN-γ production in cells cultured with rbIL15 was 10 times higher than that in cells cultured without rbIL15 (Figures-5a and b: p < 0.0001). However, the addition of mAbs against CTLA-4 and PD-L1 receptors to cultured PBMCs treated with rbIL15 did not lead to a visible increase in IFN-γ (Figure-5c).

**Figure-6:**
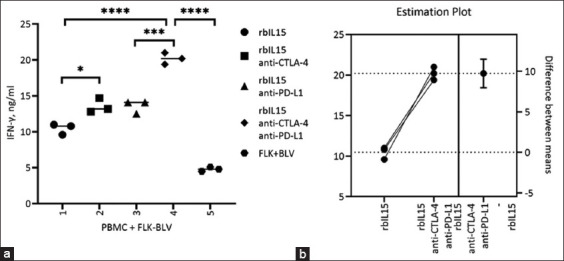
Effects of bovine BLV+ PBMC activation by combining rbIL15 with CTLA-4 and PD-L1 blockade. (a) Enzyme-linked immunosorbent assay of culture media supernatant to determine IFN-γ concentration, and (b) Estimated differences between groups. Student’s t-test was used for comparisons. At p < 0.0001, differences were considered significant. rbIL15=Recombinant bovine interleukin 15, IFN=Interferon, PBMCs=Peripheral blood mononuclear cells, CTLA-4=Cytotoxic T-lymphocyte antigen 4, PD-L1=Programmed cell death ligand 1, BLV=Bovine leukemia virus.

The potential of combining rbIL15 and mAbs against CTLA-4 and PD-L1 proteins was also explored in PBMCs from BLV+ cattle, which was a crucial aspect of this study (Figure-6). The addition of anti-CTLA-4 or anti-PD-L1 to rbIL15 did not increase IFN-γ accumulation compared with the rbIL15 + FLK + BLV group (Figure-6a). However, when both antibodies were combined with rbIL15, an increase was observed in IFN-γ production compared with that in the other groups. The triple combination resulted in an essential increase in IFN-γ production (Figure-6b).

## Discussion

This study assessed the combined effects of rbIL15 and mAbs against CTLA-4 and PD-L1 on IFN production. When the mAbs and rbIL15 were combined, no visible increase was observed in IFN-γ in PBMCs from healthy cows compared with the rbIL15 group. When PBMCs from BLV+ cows were treated similarly, this combination increased IFN-γ production compared with the other groups. PBMCs from healthy and BLV+ cows treated with rbIL15 alone did not differ in IFN production.

BLV infection is characterized by progression to leukemia, lymphocytosis, lymphoma, or lymphosarcoma. All types of leukemia are accompanied by an increase in proviral load (PVL) and an abnormal immune response because of the expansion of BLV+ cells. A study of 40% asymptomatic cows showed PVL and a decrease in specific cytokines [20]. Immune activation against viral infections involves the typical responses of specific cytokines such as IFN-γ, IL-2, and IL-4. These cytokines activate T helper 1 (Th1) and T helper 2 (Th2) cells, unlike IL-10 and TGF-β, which prevent immune activation. Researchers have found that BLV+ cows with persistent lymphocytosis had fewer Th1 and Th2 cytokines than BLV+ cows that did not have leukemia. Thus, IL-10 and TGF-β levels were higher in BLV+ cows with lymphocytosis than in those without the virus or those with low levels of these factors. The results indicate an abnormal immune response relevant to the disease’s pathogenesis. These cytokine profiles suggest that BLV causes significant immune suppression [3, 21].

IL-15 belongs to the IL-2 family and plays a significant role in both innate and adaptive immune responses. IL-15, as well as its isoform sIL-15/IL-15Rα, is considered the best cytokine to enhance the antitumor and antiviral activity of effector cells. Various forms of IL-15 may work better when combined with other immunotherapies, although they are effective in inducing tumor regression as a standalone therapy. Combining IL-15 with anti-CTLA-4 and anti-PD-L1 antibodies demonstrated highly effective antitumor effects in several cancer models. This treatment increased the counts of NK and memory CD8+ T cells. Combining IL-15 with PD-L1 and CTLA-4 blockade has been shown to improve antitumor immunity, slow tumor growth, and prolong animal survival in several preclinical studies [22, 23].

rbIL15 was used to determine the outcome in bovine PBMCs when combined with PD-L1 and CTLA-4 blockade. A prokaryotic expression system based on *E. coli* was used to produce rbIL15. The *bIL-15* gene was synthesized *de novo* after codon optimization for expression in *E. coli*. The resulting 24-kDa protein was purified by NI–NTA metal affinity chromatography. Western blotting and LC-MS/MS confirmed that the recombinant protein belonged to bIL-15. When cultured with bovine PBMCs, rbIL15 increased IFN-γ production and *STAT3*, *STAT5*, and *Bcl2* gene expression. The outcomes align with those of Vijay *et al*. [6, 19].

IL-15 influences the formation of innate and adaptive immunity and contributes to the development of various autoimmune disorders. Inflammatory bowel disease, autoimmune diabetes, rheumatoid arthritis, and celiac disease are examples of chronic inflammation and tissue-specific autoimmune diseases based on the IL-15 mechanism. As a result, chronic viral infections may upregulate IL-15 expression and support autoimmune inflammatory processes. One possible adverse effect of using IL-15 with checkpoint inhibitors in cancer immunotherapy is that autoreactive T cells may become active again [24].

The limitation of this study is that the results obtained *in vitro* are not sufficient for the practical use of the combined treatment for BLV+ cattle. Further studies are warranted to analyze T and B cells stimulated with IL-15 *in vitro* and *in vivo*. These studies will provide more information on the use of IL-15 as a molecular adjuvant to improve the intensity and duration of immune response.

## Conclusion

Recombinant bIL15 with biological activity was obtained. Stimulation of bovine PBMCs demonstrated the purified protein’s biological activity. When CTLA-4 and PD-L1 are blocked and stimulated with rbIL15, IFN-γ production increased in BLV+ cattle. The combination treatment of PBMCs from healthy cattle did not increase IFN-γ production compared with the rbIL15 group. Given the possible adverse effects, further studies are warranted to analyze the qualitative and quantitative composition of cells in IL-15-activated bovine PBMCs.

## Authors’ Contributions

KM and KT: Designed and planned the study protocol, drafted the manuscript, and data analysis. LT and ZA: Gene synthesis, cloning, and expression of recombinant bovine IL15. DK: Purification of recombinant IL-15 bovine serum and LC-MS/MS spectrometry. MN and BA: Contributed to data and sample collection, ELISA, and Western blot work. ZA and MN: Determination of recombinant bovine IL15 activity. All authors have read, reviewed, and approved the final manuscript.
